# Application of self-organizing maps to AFM-based viscoelastic characterization of breast cancer cell mechanics

**DOI:** 10.1038/s41598-023-30156-3

**Published:** 2023-02-22

**Authors:** Andreas Weber, Maria dM. Vivanco, José L. Toca-Herrera

**Affiliations:** 1 Institute of Biophysics, Department of Bionanosciences, University of Natural Resources and Life Sciences (BOKU), Vienna, Austria; 2 CIC bioGUNE, Basque Research and Technology Alliance, BRTA, Technological Park of Bizkaia, Derio, Spain

**Keywords:** Biophysics, Cancer, Cell biology, Soft materials, Computational biology and bioinformatics

## Abstract

Cell mechanical properties have been proposed as label free markers for diagnostic purposes in diseases such as cancer. Cancer cells show altered mechanical phenotypes compared to their healthy counterparts. Atomic Force Microscopy (AFM) is a widely utilized tool to study cell mechanics. These measurements often need skilful users, physical modelling of mechanical properties and expertise in data interpretation. Together with the need to perform many measurements for statistical significance and to probe wide enough areas in tissue structures, the application of machine learning and artificial neural network techniques to automatically classify AFM datasets has received interest recently. We propose the use of self-organizing maps (SOMs) as unsupervised artificial neural network applied to mechanical measurements performed via AFM on epithelial breast cancer cells treated with different substances that affect estrogen receptor signalling. We show changes in mechanical properties due to treatments, as estrogen softened the cells, while resveratrol led to an increase in cell stiffness and viscosity. These data were then used as input for SOMs. Our approach was able to distinguish between estrogen treated, control and resveratrol treated cells in an unsupervised manner. In addition, the maps enabled investigation of the relationship of the input variables.

## Introduction

Mechanical forces are important for cell migration, interaction of cells with their environment, tissue morphogenesis and in various forms of disease^1–6^. In cancer, due to deposition of aligned extracellular matrix proteins solid, stiff tumours can be recognized on the macroscale by palpation and similar techniques^7^. Interestingly, on the single cell scale, cancer cells have been found to exhibit a softer, more fluid like phenotype^8^. This change is conserved over many types of cancer tissue origin, ranging from breast, brain, prostate, kidney to lung cancer. Besides other biochemical alterations in cancer cell metabolism, they are thought to have a distinctly reorganized cytoskeletal network which contributes to their mechanical phenotype^9^. This includes altered actin cytoskeleton dynamics such as large lamellar protrusions and actin-rich micro-spikes, or altered microtubule dynamics leading to amoeboid like increased cell motility. In addition, the softening of cells appears to correlate with the level of aggressiveness, as well as changes in the adhesive and motile properties of these cells^10–12^. Therefore, the mechanical phenotype of cancer cells is thought to be a promising, label-free biomarker^13–16^. Different studies have provided evidence for the softening of cancer cells and tissue, as well as enabled the differentiation of cancer progression in tissue sections^11,17,18^.

Breast cancer is the most commonly diagnosed cancer and the first cause of death from cancer in women worldwide^19^. A common therapeutic approach in hormone-dependent breast cancer, the most common type of breast cancer, is to target estrogen receptor (ER) signalling with drugs such as tamoxifen or aromatase inhibitors. ER signalling is widely recognized in playing active roles in cytoskeleton, motility and adhesion protein expression with multiple downstream targets^20,21^. A softening of breast cancer cells with increased aggressiveness has been shown in recent works^18,22–24^. The alterations in the mechanical phenotypes of breast cancer cells treated with drugs that either inhibit or activate ER were studied in vitro^25,26^.

Mechanical properties of cells and tissue can be measured either by active or passive methods^27,28^. The first include methods such as parallel plate rheometry^29^, optical and magnetic tweezers^30,31^, micropipette aspiration^32^, optical stretcher^33^, magnetic twisting cytometry^34^ and AFM^35^, while the latter include particle tracking, and spectroscopic methods^27^. AFM is a widely applied technique, enabling imaging with nanometric precision while measuring mechanical properties at a wide set of forces, strains, and frequencies. In AFM measurements, the probe (e.g., a micrometric particle or a nanometric tip) is brought into contact with the sample and the bending of the cantilever is measured. The bending in relation to the position and stiffness of the cantilever can be converted to determine the deformation of a sample under a given force (force-distance-curves). In addition, force and deformation can be monitored over time (force-time-curves). The mechanical properties of the sample can then be determined by fitting various mechanical models of different complexity to the data^36^. There is the notion that AFM based single cell and tissue mechanics could be applied in clinical settings to support medical professionals in classification of samples such as cancer progression through evaluation of biopsies^18,37^.

Recently, machine learning (ML) and neural network algorithms have been applied to surface probe microscopy data^38^, including image segmentation^39^, automatic data processing^40–43^, cancer cell classification and progression grade evaluation^44–48^. These approaches were performed in a supervised way, with manual classification of a data set to train the ML algorithms that were applied to classify data. In this work, we apply self-organizing maps (SOMs, also called Kohonen maps) as an artificial neural network approach to analyse mechanical measurements of breast cancer cells^49–52^. SOMs are unsupervised artificial neural networks that enable 2D visualization of multidimensional data space while still conserving the topology of the dataset. They have been widely applied in data science^51^, speech and document processing^53^, and recently also in life sciences and chemometrics^54^. One result of SOMs are U-matrix plots that show the similarity/dissimilarity between neurons, enabling post hoc cluster analysis^51^.

In this work we have used epithelial breast cancer (MCF-7) cells as model for breast cancer cell mechanics and performed stress relaxation measurements in the nuclear regions of cells via AFM using sharp pyramidal tips. We then applied a viscoelastic model to derive mechanical parameters that were used as input layer for a SOM, which was followed by cluster analysis. We show the principal ability of SOMs to differentiate between control, estrogen and resveratrol treated cells, while the SOMs placed control and tamoxifen treated cells in close neighbourhood.

## Results

### Estrogen receptor interacting drugs change breast cancer viscoelastic properties

Table 1 shows the values derived from the 5-element Maxwell model fitting for the stress relaxation segments of the cells measured via AFM after the three different treatments (plus the carrier DMSO, as control). Control cells showed moduli in the range of a few hundred Pa and relaxation times of 0.2 and 3.1 s respectively. Treatment with estrogen led to significant softening and less viscous mechanical phenotype, comparable to other published results (reduction of moduli of around 50%)^26,55^. The treatment with tamoxifen did not lead to significant changes in the mechanical properties of the cells, according to this analysis (increase of 10 to 15 % in moduli and viscosities). Finally, treatment with resveratrol led to a stark increase of moduli of up to 6 times, slightly longer relaxation times and threefold increased viscosities. These data overall agree well with published literature^25^. Note that some of the parameters showed strong correlations, as moduli reflect overall the elastic properties of the samples and are inverse proportional to indentations, while in the used model, viscosities are proportional to relaxation times and moduli.

**Table 1:** Viscoelastic properties of the different groups of treated cells (control (CTL), estrogen (E2), tamoxifen (TAM) and resveratrol (RESV)) derived from the stress relaxation measurements.

	Control	E2 (100 nM)	TAM (5 µM)	RESV (50 µM)
${\varvec{\delta}}\boldsymbol{ }[{\varvec{\upmu}}\mathbf{m}]$	2.11 ± 0.05	2.82 ± 0.05	2.04 ± 0.04	1.32 ± 0.05
${{\varvec{E}}}_{\boldsymbol{\infty }}\boldsymbol{ }[\mathbf{P}\mathbf{a}]$	444 ± 23	187 ± 11	510 ± 25	2959 ± 235
${{\varvec{E}}}_{1\boldsymbol{ }}[\mathbf{P}\mathbf{a}]$	299 ± 14	150 ± 7	346 ± 16	1046 ± 77
${{\varvec{E}}}_{2}[\mathbf{P}\mathbf{a}]$	289 ± 14	142 ± 7	320 ± 17	1024 ± 70
${{\varvec{E}}}_{{\varvec{i}}{\varvec{n}}{\varvec{s}}{\varvec{t}}}\boldsymbol{ }[\mathbf{P}\mathbf{a}]$	1031 ± 47	479 ± 23	1175 ± 54	5029 ± 367
${{\varvec{\tau}}}_{1}\boldsymbol{ }[\mathbf{s}]$	0.19 ± 0.01	0.19 ± 0.01	0.17 ± 0.01	0.19 ± 0.01
${{\varvec{\tau}}}_{2}\boldsymbol{ }[\mathbf{s}]$	3.13 ± 0.14	2.93 ± 0.10	3.15 ± 0.15	3.23 ± 0.16
${{\varvec{\eta}}}_{1}\boldsymbol{ }[\mathbf{P}\mathbf{a}\mathbf{s}]$	56 ± 3	27 ± 1	58 ± 3	187 ± 14
${{\varvec{\eta}}}_{2}\boldsymbol{ }[\mathbf{P}\mathbf{a}\mathbf{s}]$	885 ± 55	403 ± 22	1052 ± 80	3198 ± 253

The values show mean value ± standard error of mean. $\delta$ is indentation, ${E}_{\infty }$ the equilibrium modulus, ${E}_{1}$ and ${E}_{2}$ the moduli of the springs in Maxwell arms, ${E}_{inst}$ the instantaneous modulus, ${\tau }_{1}$ and ${\tau }_{2}$ the relaxation times of the dashpots and the viscosities are ${\eta }_{1}$ and ${\eta }_{2}$. N is 412 for CTL, 224 for E2, 229 for TAM and 171 for RESV.

### Training self-organized maps with viscoelastic cell properties

**Figure 1:**
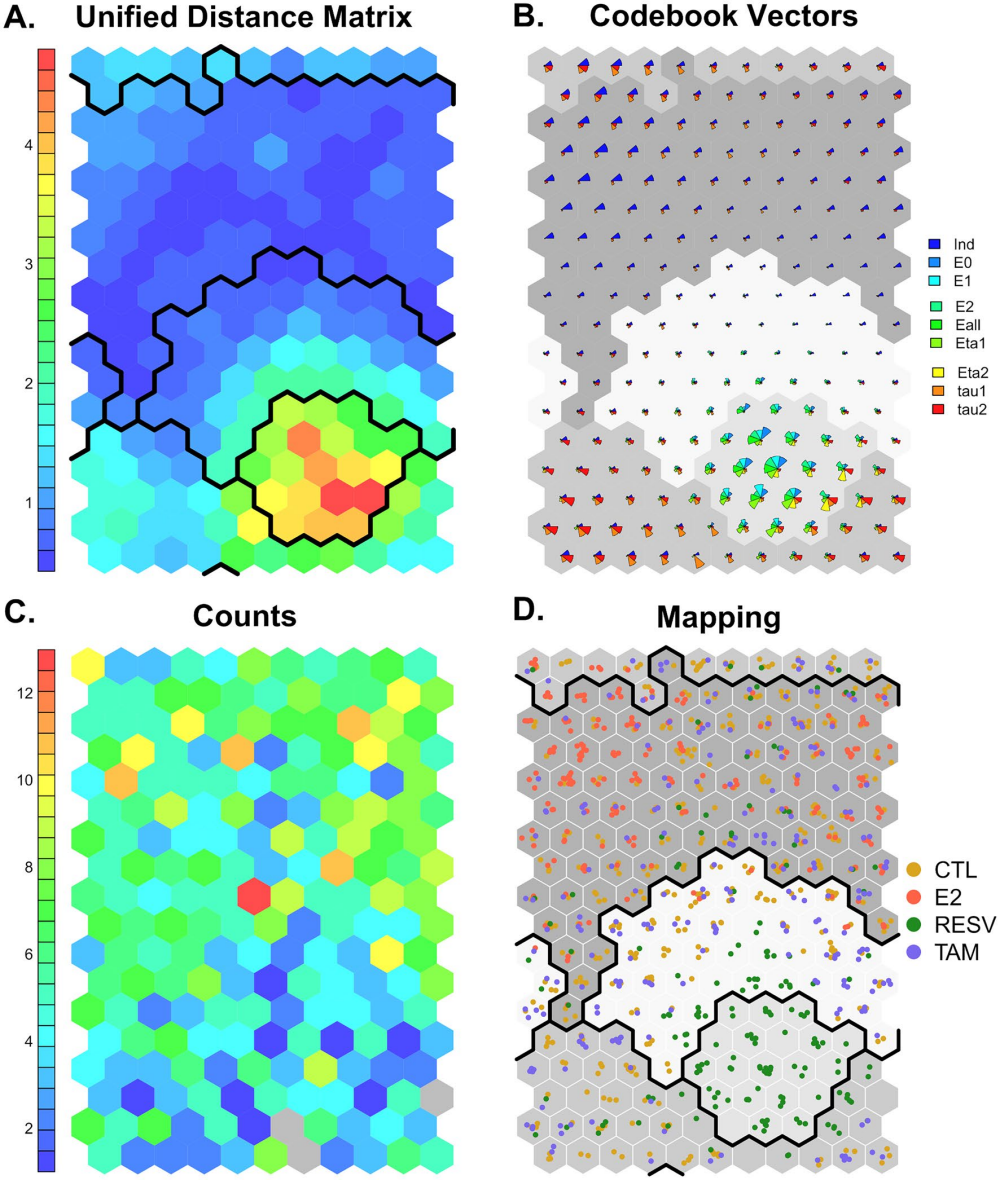
Results of training the self-organizing map using a batch algorithm. (**A**) Unified distance matrix plot. The colour scale corresponds to Euclidean distance between nodes. The post hoc clustering was performed using kmeans clustering with 4 centroids. (**B**) Fan-diagram showing the spatial distribution of the nine different variables on the 2D map. $\delta$ is indentation, ${E}_{\infty }$ the equilibrium modulus, ${E}_{1}$ and ${E}_{2}$ the moduli of the springs in Maxwell arms, ${E}_{inst}$ the instantaneous modulus, ${\tau }_{1}$ and ${\tau }_{2}$ the relaxation times of the dashpots and the viscosities are ${\eta }_{1}$ and ${\eta }_{2}$. (**C**) Counts plot of the number of observations per node. In grey nodes, zero observations are placed. (**D**) Mapping of the treatment as input factors. Cluster 1 includes 227 measurements, Cluster 2 69, Cluster 3 195 and Cluster 4 545.

Self-organizing maps were trained with the scaled cell viscoelastic properties (centred and divided by the standard deviation) obtained after the different treatments^56^. The performance of both iterative and batch algorithms was compared using a wide range of map parameters (iteration steps, size and shape of map, learning rates, and distance functions). These calculations were repeated multiple times to account for randomness in the initialisation and training. The maps shown from here on are the results of a batch trained map with 11 times 18 hexagonal nodes, a toroidal topography, a gaussian neighbourhood function, learning rates of (0.05, 0.01), Euclidean distance functions and 1000 iteration steps (map with smallest quantization and topographic error). Figure 1 shows the results of the SOM training.

In Fig. 1A the unified distance matrix of the SOM can be seen. The colour scale indicates the Euclidean distance between nodes, blue tones indicate small distances, while red tones indicate large ones. A distinct region with high dissimilarity can be observed at the bottom right of the map (visible as cluster). Note that the map is toroidal, thus reducing edge effects. The dark black lines indicate the post hoc clustering of the nodes. Figure 1B shows the distributions of the single codebook vectors and the determined clusters in greyscales. This analysis can be used to analyse the distribution of the single input variables. Even for the low number of input variables, this analysis can be quite overwhelming, as only regions with significant differences can be seen easily. As an example, the nodes that showed high dissimilarity in Fig. 1A on the right bottom side of the map showed large values of all moduli (large green and cyan fans), while only possessing small indentations of around 1 μm (small dark blue bars) and small relaxation times (red and orange bars) in the fan diagram in Fig. 1B. Measurements with high indentation values (2.5–3 μm) appeared to be placed in vicinity on the top left-hand side of the map. Figure 1C shows the number of measurements that were placed in each node. Finally, Fig. 1D uses the treatment factor (as these are known from the input data) and visualizes where measurements of respectively treated cells were placed on the map. The cluster on the bottom right side of the map is made up solely of resveratrol treated cells, while a large amount of estrogen treated cell measurements are located at the top left side of the map. Note that the number of clusters was defined as 4 to reflect the different treatments.

### Resveratrol and estrogen treated cells can be distinguished by unsupervised SOMs

Tables 2 and 3 show a further analysis of the four clusters. Cluster 1 was predominantly (80%) made of cells from the control group (104) and tamoxifen treated cells (77). Cluster 2 only included resveratrol treated cells. Cluster 3 was a mixture of all cell types, mostly control and tamoxifen treated ones (50% control, 7% E2, 16% RESV and 27% TAM). Finally, cluster 4 was made up mostly of control and estrogen treated cells (39% control, 37% E2, 6% RESV and 18% TAM). With respect to mechanics, cells placed in cluster 1 and 3 showed intermediate stiffnesses and indentations. Cluster 3 represented higher relaxation times than all other clusters. Resveratrol treated cells, located in Cluster 2, presented high stiffness, low indentations, high viscosities, and intermediate relaxation times. The characteristics of cluster 4 were very soft cells, with large indentations, low moduli, intermediate relaxation times and low viscosities. The cluster analysis shows that resveratrol treated cells can be readily distinguished from the other groups, while estrogen treated cells could mostly be found in a cluster together with control and tamoxifen treated cells. Control cells and tamoxifen treated cells co-localised on the maps and the SOMs were not able to distinguish between them. This was expected, as the mechanical properties were very similar and the only used input for the neural network. Additional input parameters would be needed to distinguish between these treatments.

**Table 2:** Analysis of the four cluster with respect to kind of treatment.

Cluster	N	N (CTL)	N (E2)	N (RESV)	N (TAM)
1	227	104	7	39	77
2	69	0	0	69	0
3	195	98	13	32	52
4	545	210	204	31	100

**Table 3:** Analysis of the four cluster with respect to the determined viscoelastic properties. The values shown are mean values ± the standard error.

	Cluster 1	Cluster 2	Cluster 3	Cluster 4
${\varvec{\delta}}\boldsymbol{ }[{\varvec{\upmu}}\mathbf{m}]$	1.60 ± 0.02	0.84 ± 0.02	2.04 ± 0.04	2.52 ± 0.05
${{\varvec{E}}}_{\boldsymbol{\infty }}\boldsymbol{ }[\mathbf{P}\mathbf{a}]$	987 ± 69	5348 ± 207	537 ± 45	275 ± 13
${{\varvec{E}}}_{1\boldsymbol{ }}[\mathbf{P}\mathbf{a}]$	510 ± 19	1850 ± 68	352 ± 15	188 ± 7
${{\varvec{E}}}_{2}[\mathbf{P}\mathbf{a}]$	498 ± 19	1773 ± 55	351 ± 20	175 ± 8
${{\varvec{E}}}_{{\varvec{i}}{\varvec{n}}{\varvec{s}}{\varvec{t}}}\boldsymbol{ }[\mathbf{P}\mathbf{a}]$	1996 ± 95	8971 ± 294	1240 ± 74	638 ± 26
${{\varvec{\tau}}}_{1}\boldsymbol{ }[\mathbf{s}]$	0.13 ± 0.01	0.18 ± 0.01	0.27 ± 0.01	0.17 ± 0.01
${{\varvec{\tau}}}_{2}\boldsymbol{ }[\mathbf{s}]$	2.21 ± 0.08	3.13 ± 0.13	5.27 ± 0.14	2.71 ± 0.09
${{\varvec{\eta}}}_{1}\boldsymbol{ }[\mathbf{P}\mathbf{a}\mathbf{s}]$	71 ± 4	322 ± 14	94 ± 4	32 ± 2
${{\varvec{\eta}}}_{2}\boldsymbol{ }[\mathbf{P}\mathbf{a}\mathbf{s}]$	1124 ± 60	5291 ± 232	1859 ± 133	477 ± 27

### SOMs for exploration of mechanical property interconnectivity

**Figure 2:**
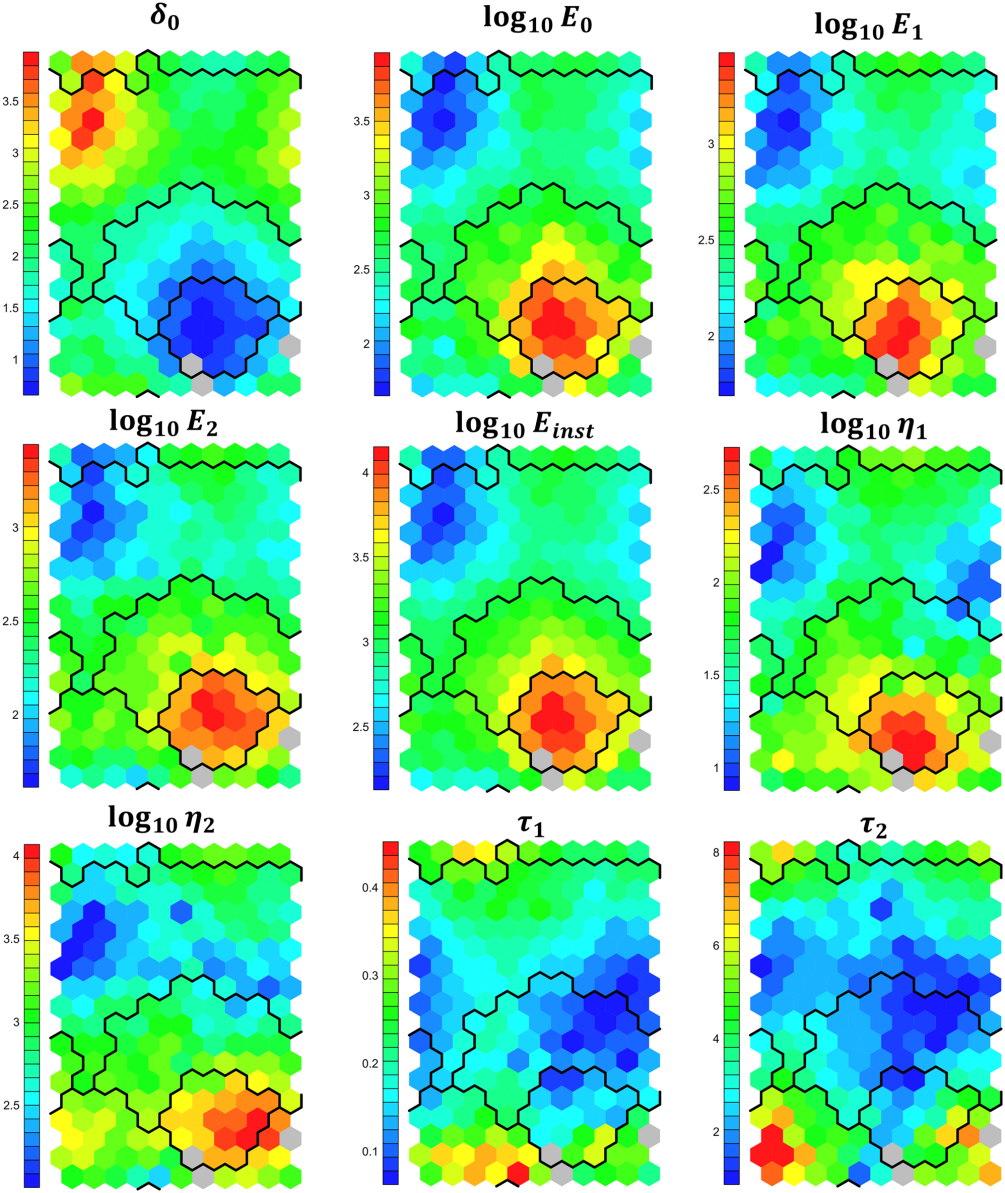
Component maps of the nine input parameters after training the SOMs. The colour coding indicates the magnitude of the nodes. The moduli and viscosities are logarithmically scaled to allow for comparison. The black lines show the borders of the four calculated clusters. Grey nodes have no measurements placed in them.

One strength of self-organizing maps is that they allow exploration of interconnectivity of different input variables. Figure 2 shows the component maps of the 9 input variables after training. In addition, the cluster analysis was superimposed on the maps. Note that the moduli and viscosities were plotted in logarithmic scale to allow for better comparison.

A correlation between all moduli and the viscosities was observed. In addition, the six parameters ${E}_{\infty }$, ${E}_{1}$, ${E}_{2}$, ${E}_{inst}$, ${\eta }_{1}$ and ${\eta }_{2}$ were negatively correlated with the indentation (red colour tones indicating high values while the indentation shows blue colour tones indicating low values). This fits together with the mechanical model, as cells that are softer should show lower moduli, higher fluidity (lower viscosity) and larger deformations. Two distinct regions of deformation can be seen in the component maps: On the left top, a region with high indentation and low stiffness as well as viscosity was found, which corresponded mostly to estrogen treated cells (comparison of Figs. 2 and 1D). On the other hand, an island of low indentation, large moduli and large viscosities was detected on the bottom right part of the maps, which mostly displayed measurements made on resveratrol treated cells. Regarding the relaxation times, a large patch of small relaxation times was identified in the vertical middle region of the maps. Interestingly, these values appeared to correspond to intermediate deformation, moduli and viscosity values. A small patch of long relaxation times was noticed at the bottom left of the maps, which represented intermediate stiffness and deformation values.

## Discussion

In this work we apply self-organizing maps to viscoelastic data of breast cancer cells derived from stress relaxation atomic force microscopy measurements after treatment with different ER interacting drugs. We employ an unsupervised approach and use the output layer of this artificial neural network for cluster analysis. We show that resveratrol treatment leads to stiffening and higher viscosities, estrogen treatment to a softening and fluidization, while tamoxifen treatment does not appear to significantly affect these mechanical properties. We then provide evidence that this type of analysis can potentially be used to classify cells according to treatment in an unsupervised manner.

Cancer cells show altered mechanical phenotypes compared to non-malignant counterparts^16^. This results among other factors from changes in the mechanics of the environment, tumour hypoxia and aberrant gene expression, altering signalling pathways that affect cell metabolism, motility, and adhesion. Targeting the mechanical adaptation programme of cancer as part of therapy has been proposed, although its wide implications in cell function may lead to undesired side effects. Potential targets include disruption of actin filament assembly and dynamics, inhibition of myosin or targeting Rho/ROCK pathways, among others. Different drugs used as therapy such as paclitaxel, cisplatin, doxorubicin, and 5-fluorouracil, have been investigated for their effects on mechanical properties of cells from various origins. These treatments mostly led to cell stiffening, which contributed to altered cytoskeletal dynamics resembling a reversal of the epithelial to mesenchymal transition^57–59^. Such changes are believed to lead to reduced cell migration, partly inhibiting cancer growth. We provide further evidence that resveratrol leads to stiffening of cells, with novel data regarding the viscoelastic properties, while estrogen leads to a softening and fluidization of the cells. Interestingly, tamoxifen does not appear to significantly alter the mechanics of MCF-7 cells on solid substrates. A more thorough analysis regarding cytoskeletal arrangements and protein expression patterns resulting from such treatments is needed.

In the present approach we have used viscoelastic data derived from stress relaxation measurements using a five-element Maxwell model fitting. We have omitted the use of any further characteristics of force spectroscopy curves on cells (such as hysteresis, work of adhesion, tethers, tension). In addition, we have decided to ignore the measurement data itself, but rather evaluate derived mechanical properties. A critical parameter to consider is the computational time the analysis takes. While the computation of the self-organizing map approach chosen is relatively straightforward, the data processing steps to calculate the viscoelastic properties from raw force-distance-curves is the time intensive step of this analysis (the used framework takes around 5 s for curve processing, fitting, and property calculation per curve). Using simpler models (such as only elastic properties, indentations at a given force, hysteresis, adhesion properties) one can probably reduce the computational costs. In addition, we show that most of the variables used as inputs are correlated. Therefore, a priori reduction of data dimensionality using principal component analysis followed by statistically relevant principal components as input for the SOMs, will also speed up the analysis.

Most of the neural network or machine learning approaches that have been applied to AFM force spectroscopy data on biological materials are based on supervised methods. Recently, three different approaches have been provided in the literature: Using machine learning to classify the quality of curves^41^, training a machine learning algorithm with curve shapes^44^, or using curve characteristic properties as inputs for supervised training^43,45,60^. Such approaches have shown promising results in correctly classifying cancer cells from healthy cells, as well as cancerous tissue. Compared to these approaches, SOMs arguably show strengths in data exploration, enabling the simplification of a multidimensional data space to 2D representations. In addition, SOMs can also be performed supervised. With respect to the ability of this SOM approach to identify the treatment of breast cancer cells, we report mixed results. The addition of further independent variables, including more measurements, and morphological characteristics of the cells will probably help in training a SOM algorithm to successfully classify the different types of cells unsupervised.

## Materials and methods

**Figure 3:**
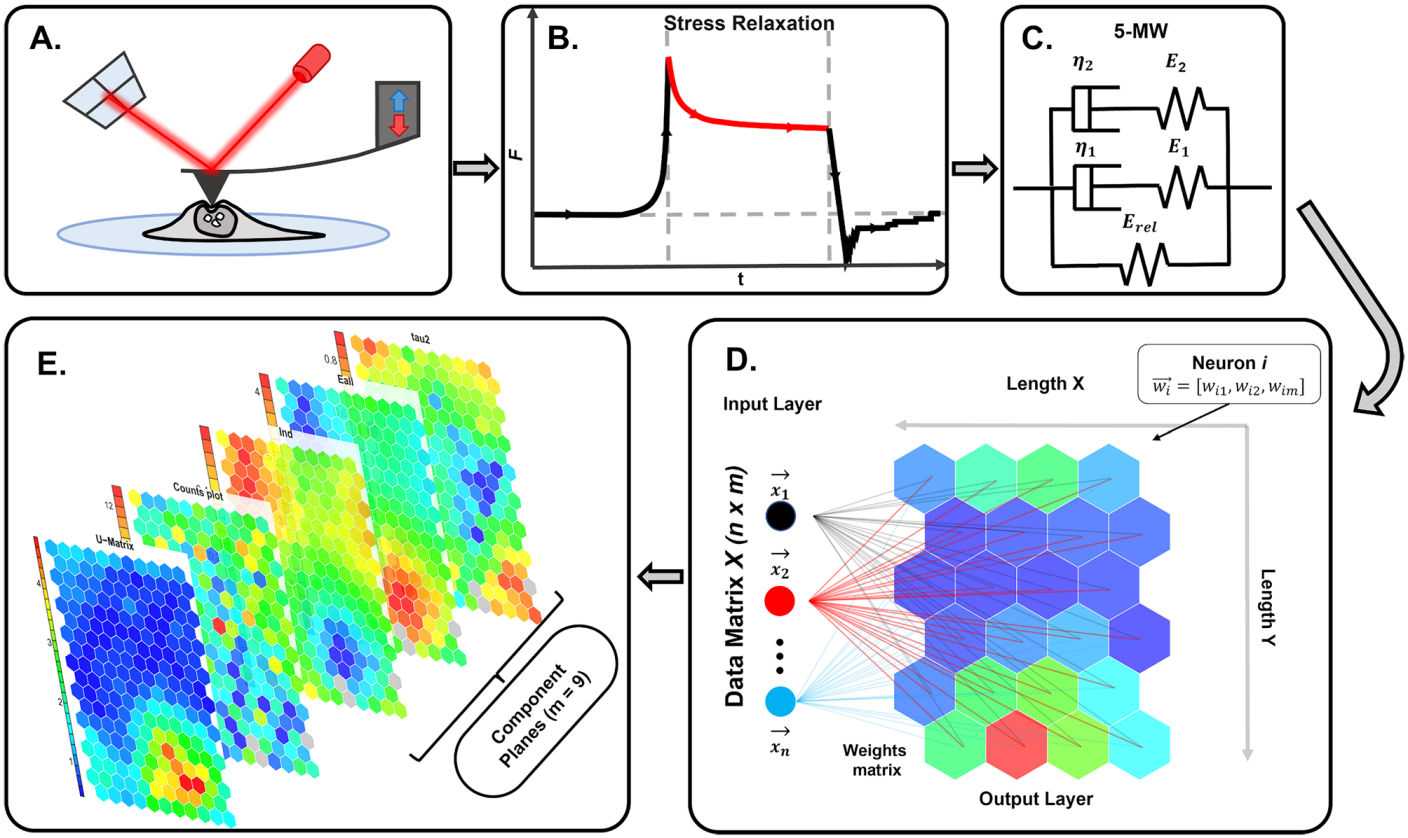
Methodology of the present study. (**A**) Cell viscoelastic properties are measured using stress relaxation experiments with AFM. (**B**) and (**C**). The resulting stress relaxation segment (force decay over time while keeping the deformation constant) is fitted using a five-element Maxwell model. This results in 9 input variables. (**D**) A self-organizing map is trained unsupervised. (**E**). The complex interconnectivity of the data can be represented in 2D maps, allowing for data exploration.

Figure 3 shows the overall methodology performed in this study. Briefly, in step 1, cell mechanical properties were quantified using stress relaxation measurements via AFM. A scheme of such analysis can be seen in Fig. 3B. A 5-element Maxwell model was fitted to the stress relaxation curves, resulting in 9 fitting parameters (Fig. 3C). These mechanical parameters were used to train SOMs (Fig. 3D). Finally, these SOMs were used to visualize the result of the artificial neural network. To test the ability of the trained aNNs to distinguish between cells with different mechanical properties, MCF-7 breast cancer cells were treated with different substances used in breast cancer therapy that are known to influence cell mechanical properties.

### Cell mechanical measurements

MCF-7 cells were obtained from the American Type Culture Collection (ATCC) and grown as previously described^61^. Cells were seeded at a concentration of 200,000 cells/mL on plasma-treated glass slides in DMEM medium supplemented with 10% fetal bovine serum (FBS) and 1% penicillin/streptomycin at 37 °C in 5% CO_2_. Cells were either treated for 48 hours with DMSO (0.05 %), 100 nM estrogen (E2), 50 µM resveratrol (RESV) or 5 µM tamoxifen (TAM). For mechanical measurements, a JPK Nanowizard III with a CellHesion extension was used. Pyramidal cantilevers (DNP-S, B, Bruker), with nominal stiffness of 0.12 N/m, a resonance frequency of 23 kHz in air, an opening angle of 22° and a nominal tip radius of 10 nm were used. Calibration by thermal noise making use of the equipartition theorem was performed for each cantilever^62^. Measurements were done with a constant approach and retract rate of 5 µm/s, a maximum load of 1 nN, curve lengths of 50 µm, a constant deformation 10 s pause segment at an initial load of 1 nN and a sampling rate of 1024 Hz. Measurements were done in L15 medium at 37 °C and performed in the central region of the cell above the nucleus.

### Data evaluation

Data was extracted using the JPKSPM software (JPK, Bruker), and all further steps were performed in R. Data pre-processing was done making use of the R afmToolkit, a package for AFM force-distance and force-time analysis developed by our group^63–65^. Briefly, contact and detachment points were calculated, the curves were corrected for their baselines and the sample deformation $(\delta )$ was determined. In the present analysis, only the stress relaxation segments were considered. Those were fitted with a five-element Maxwell model as

$$E\left( t \right) = E_{\infty } + E_{1} e^{{ - {\raise0.7ex\hbox{$t$} \!\mathord{\left/ {\vphantom {t {\tau_{1} }}}\right.\kern-0pt} \!\lower0.7ex\hbox{${\tau_{1} }$}}}} + E_{2} e^{{ - {\raise0.7ex\hbox{$t$} \!\mathord{\left/ {\vphantom {t {\tau_{2} }}}\right.\kern-0pt} \!\lower0.7ex\hbox{${\tau_{2} }$}}}} ,$$

Where $E\left( t \right)$ is the relaxation modulus, $E_{\infty }$ the equilibrium modulus, $E_{1}$ and $E_{2}$ the moduli of the springs in Maxwell arms and $\tau_{1}$ and $\tau_{2}$ the relaxation times of the dashpots. The viscosity $\eta_{i}$ of the dashpots is defined as

$$\eta_{i} = E_{i} \tau_{i}$$

### Self-organizing maps

Self-organizing maps were first introduced by Kohonen and have since received more and more attention in wider fields of statistics, chemometrics and life sciences. Their major use is found in data exploration and visualization, as they enable the breakdown of complex data matrices and the interaction of components via 2D similarity representations. In addition, the representation keeps the topological information of the input data. Here we describe the principles of the classical iterative and the batch algorithms. Any reader is referred to the extensive reviews on the theoretical backgrounds, application and usage of SOMs in statistical analysis, life sciences and industry^51,52,66^. Such applications are wide ranged, including analysis of spectroscopy data^67,68^, automated feature extraction from microscopy images^69^, segmentation and grading of tumours or application to omics data^70,71^.

SOMs are artificial neural networks that use unsupervised training procedures (competition, cooperation, and adaptation). In principle, a (mostly) 2D network of neurons (nodes, codebooks) is set up that is trained to represent the distance structure of the input data as closely as possible (see Fig. 3D). In the case of SOMs, nodes are defined by codebook vectors (weights) that encompass all variables of the input data. Prior to training, the network is initialized either randomly or linearly. One needs to a priori define network properties such as topology (hexagonal or rectangular), architecture of the grid (dimensions, shape, repetitiveness, dynamically growing SOMs), number of neurons (depends on input data, approach, and analysis route). Depending on the type of distance measure applied, input data needs to be normalized to account for different scaling.

In the original iterative approach, the following training steps are performed:

1. Randomly select an input vector.
2. Calculate the distance between the input and all neurons in the network (using Euclidean distance after normalization works for most cases).
3. Determine the best matching unit (BMU) with the lowest distance to the input vector (competition).
4. Calculate the neighbouring neurons that will also be updated according to the chosen neighbourhood function (gaussian, bubble) (cooperation).
5. Update the BMU and the neighbours applying learning rates, by moving them closer to the input vector (adaptation).
6. Repeat steps 1 to 6 until a given number of iterations is reached or the system converges to stability.

Both the neighbourhood distance and the learning rate are decreasing monotonically with each iteration. The learning rate is adjusted depending on the proximity of the neuron to the BMU. The learning phase is split in two main segments, first with a high learning rate (ordering phase) and then with a lower one (convergence phase). In batch SOMs, a similar approach is performed but rather all neurons are compared with all input data.

### Application of SOMs to breast cancer viscoelastic properties

As input data, we used the 9 properties ($\delta , { E}_{\infty }, { E}_{1}, { E}_{2}, {E}_{inst}, {\tau }_{1}, {\tau }_{2}, {\eta }_{1}, {\eta }_{2})$ derived from the fitting of the stress relaxation segments. Outliers were removed and data was normalized by mean value and standard deviation. For SOM calculations, the R package Kohonen was used^56^. Rectangular, toroidal maps with hexagonal nodes were used. The x-y-ratio of the maps was determined from the relationship of the eigenvalues of the first two principal components of the data. A Gaussian neighbourhood function and Euclidean distance functions were utilized. The learning rate was set to 0.05 in the beginning and decreased to 0.01. The neighbourhood function was defined as exponential decay starting with a value that covered 2/3 of all unit-to-unit distances. For the classical, iterative approaches, 50,000 iterations were performed, the calculations were repeated 50 times and the map with the lowest quantization error was chosen. Similarly, batch SOMs were performed with 1000 iterations and repeated 50 times. The results shown in this manuscript correspond to the batch SOM with the lowest quantization error.

## Data Availability

Data is available per request from the corresponding author.
